# Graphene-Based Composite Membrane Prepared from Solid Carbon Source Catalyzed by Ni Nanoparticles

**DOI:** 10.3390/nano11123392

**Published:** 2021-12-14

**Authors:** Jing Li, Jialiang Liu, Jinshui Liu, Jinfeng Lai, Yuxun Chen, Wenjun Li

**Affiliations:** 1 School of Chemistry and Chemical Engineering, South China University of Technology, Guangzhou 510641, China; ljing@scut.edu.cn (J.L.); 201920122396@mail.scut.edu.cn (J.L.); 202020124091@mail.scut.edu.cn (J.L.); laijinfeng666@126.com (J.L.); 2 SCUT-Zhuhai Institute of Modern Industrial Innovation, Zhuhai 519175, China; 3 School of Mechanical and Automotive Engineering, South China University of Technology, Guangzhou 510641, China; 4 Beijing Headquarters of Space Vehicle, Beijing 100086, China

**Keywords:** oxidized graphene, thermal conductivity, hydroxypropyl methyl cellulose, Ni nanoparticles

## Abstract

Emerging as a light, flexible and highly thermally conductive material, graphene-based membranes have attracted extensive attention in thermal management field. However, the preparation of high-quality graphene-based membranes usually involves complex processes and thermal annealing at ultra-high temperature, which limits their large-scale application in thermal management field. In our study, reduced graphene oxide-Ni-hydroxypropyl methyl cellulose (RGO-Ni-HPMC) composite membrane was prepared from catalytic pyrolysis of hydroxypropyl methyl cellulose (HPMC) with Ni nanoparticles to generate multilayer graphene and form phonon transport channels between adjacent graphene layers. Further, our study shows that the RGO-Ni-HPMC composite membrane has a good heat dissipation effect at the hot spots at high temperature. The average temperature of hot spots is reduced by 11.5 °C. It is expected to solve the heat dissipation problem of high-power electronic equipment.

## 1. Introduction

With the rapid development of electronic assembly technology, the large-scale application of miniaturized, integrated and high-frequency high-power electronic equipment also requires more and more effective heat dissipation upon operation [1,2,3]. At present, copper, aluminum and other traditional radiators are widely used in electronic equipment. However, metal materials have disadvantages such as poor flexibility and high specific weight. On the one hand, it increases the total weight of the heat dissipation system, which is not conducive to the development of devices with light weight and miniaturization. On the other hand, the difficulty of stretching and bending metals also limits their use in complex heat dissipating components, such as foldable smart phones, laptop computers and spacecraft, etc. [4]. In addition, due to the limitation of its own properties, for example the thermal conductivity of copper foil is only 397 W/(m·K) and that of solid aluminum is only 237 W/(m·K), metal materials are not suitable for higher heat flux applications [5]. Therefore, in order to meet the higher heat dissipation demand of electronic equipment, it has become an urgent need to develop a kind of light and flexible heat dissipation material with higher thermal conductivity.

Although the graphite film assembled by graphite sheet (such as expanded graphite film, etc.) also has high thermal conductivity, its mechanical properties come from the friction between graphite sheet layers after pressing treatment, resulting in poor flexibility [6]. Graphene is a two-dimensional carbon material with hexagonal honeycomb lattice composed of carbon atoms with sp^2^ hybrid orbitals [7]. It has the advantages of ultra-high carrier mobility, good mechanical properties, excellent heat conduction characteristics and optical properties. The thermal conductivity of single-layer graphene without defects can be as high as 5300 W/(m·K), it is significantly higher than that of currently known thermally conductive materials, such as copper, silver, diamond, carbon nanotubes, etc. [8,9] Therefore, the two-dimensional macroscopic material assembled by graphene has the advantages of high thermal conductivity and good flexibility, and is considered to be a heat dissipation material with great development space. For example, Huang et al. [10] prepared GO films by roller coating with GO dispersion as the precursor, and then conducted graphitization thermal reduction at high temperature to obtain a graphene-based thermally conductive membrane with good flexibility. When the thermal reduction temperature was 2800 °C, the planar thermal conductivity of the film was 826.0 W/(m·K). Although the graphene assembly membrane prepared by Huang et al. has excellent performance, its production process involves graphitization at a high temperature above 2000 °C, which requires expensive production equipment, has a complex preparation process, huge energy consumption and high cost, which greatly limits its application in high-power electronic equipment.

Therefore, in order to promote the further development and application of flexible graphene based membrane with high thermal conductivity, many researchers have done a lot of research work in reducing its production cost, such as replacing the high-temperature graphitization process (below 1000 °C) with the modest thermal reduction process (above 2800 °C), etc. For example, Jin et al. prepared GO films with vacuum pumping and filtration method, and then processed the film with a 600 °C modest thermal reduction process to obtain the thermal conductivity of graphene-based thermally conductive membrane, which was 373 W/(m·K) [11]. Hou et al. obtained graphene nanosheets with liquid phase stripping method and assembled them with vacuum filtration to obtain graphene-based membrane containing a small amount of oxygen-containing groups [12]. Finally, a graphene-based thermally conductive film with good flexibility was obtained with thermal annealing at 1060 °C to remove the residual oxygen-containing groups, with a thermal conductivity of 220 W/(m·K). Although the modest thermal reduction process has a lower cost, the quality of the prepared graphene-based film is generally not high. Therefore, improving the thermal conductivity of the prepared graphene-based film under modest thermal reduction process condition is of great research value for realizing the large-scale application of graphene-based thermal conductivity.

Here, we proposed to use GO (Graphene Oxide) as the dominating raw material, HPMC (hydroxypropyl methyl cellulose) as the external solid carbon source, Ni nanoparticles as the catalyst, use solvent evaporation and modest hot pressing reduction process to prepare high thermal conductivity reduction RGO (Reduced Graphene Oxide)-Ni-HPMC matrix composite membrane. After thermal annealing treatment at 800 °C, Ni nanoparticles catalyzed and cracked HPMC on the surface to generate multilayer graphene and form phonon transport channels between graphene layers, thus improving the overall heat transfer rate of graphene-based composite membrane. When the mass ratio of GO, Ni and HPMC is 100:15:10, the thermal conductivity of RGO-Ni-HPMC-15 composite film reaches the maximum of 425.1 W/(m·K).The planar heat conduction test and heat dissipation experiment of simulated electronic equipment further show that the RGO-Ni-HPMC-15 composite membrane prepared in this study has excellent heat conduction performance in the plane direction and good heat dissipation and temperature control effect for high temperature equipment.

## 2. Materials and Methods

### 2.1. Experimental Material

Natural graphite powder (325 mesh), provided by Qingdao Tengshengda Carbon Machinery Co., Ltd., Qingdao, China. Concentrated sulfuric acid (H_2_SO_4_, 98 wt%), provided by Guangzhou Chemical Reagent Factory, Guangzhou, China; concentrated hydrochloric acid (HCl, 36 wt%), provided by Guangzhou Chemical Reagent Factory; potassium permanganate (KMnO_4_), provided by Guangzhou Chemical Reagent Factory; hydrogen peroxide (H_2_O_2_, 30 wt%), provided by Guangzhou Chemical Reagent Factory; hydroxypropyl methyl cellulose(HPMC, viscosity: 400 mPa·s), provided by Shanghai McLean Biochemical Technology Co., Ltd. Shanghai, China.; Ni nanoparticles (20–100 nm), provided by Shanghai McLean Biochemical Technology Co., Ltd.

### 2.2. Preparation of GO

Graphite preprocessing: weigh 5 g graphite (325 mesh) and add it to dilute HCl (volumetric ratio of condensed hydrochloric acid and water is 1:9), wash the mixture twice to remove metal impurities, filter it with filtration paper in a funnel, then neutralize the solid with a large amount of deionized water, and dry the solid in a blast drying oven at 100 °C for 8 h.

Graphite oxidation treatment: the above solid is mixed with 120 mL condensed sulfuric acid in a 500 mL triple-neck flask and placed in a circulating ice water bath at 0 °C. Keep the temperature of the mixture below 10 °C and stir it with a large mixer at 20 rpm. Add 15 g KMnO_4_ to the flask slowly for 10 times in an average period of 30 min. After adding KMnO_4_, adjust the temperature of the circulating water bath to 35 °C and keep the mixture at this temperature for 30 min. Under the protection of normal temperature water bath, transfer the mixture to 250 mL of deionized water to form in the 1000 mL beaker purple solution, then keep the temperature of circulating water bath at 85 °C, and preserve the mixture under this temperature and stir it in magnetic circumstance for 6 min, the graphite sample has been completely oxidized at this time. Finally, add 700 mL of deionized water and 20 mL of 30% H_2_O_2_ to remove excess KMnO_4_ through chemical reaction, and the solution turns out to be bright yellow.

Preparation of GO with ultrasonic exfoliation: the bright yellow solution was preserved for stratification (the volume ratio of the clear solution at upper layer and turbid solution at the lower layer was 9:1), then poured the supernatant carefully, and added 500 mL of dilute hydrochloric acid (the volume ratio of condensed hydrochloric acid and water was 1:9) to turbid solution at the lower layer to continue the motionless stratification. Then poured the supernatant, and washed the remained turbid solution at the lower layer twice. After finally removing the supernatant, added 300 mL of deionized water to the turbid solution, spin the solution in a high speed centrifuge at 8000 rpm, and removed the supernatant. Centrifugal washing was carried out repeatedly in the centrifuge until no white precipitation was detected by BaCl_2_, indicating that there was no SO_4_^2−^. Then the solid was carefully scraped into a 2000 mL beaker, and added 1000 mL water to it, after that stirred the solution in ultrasonic circumstance for 30 min. The solution was distributed into 6 dialysis bags and soaked in 2000 mL of deionized water for dialysis treatment which lasted for a week and the deionized water was changed every other day. After a week of dialysis treatment, the solution in the dialysis bag was transferred to a 2000 mL beaker and an appropriate amount of deionized water was added to prepare GO dispersible solution with a concentration of 5 mg/mL.

**Figure 1:**
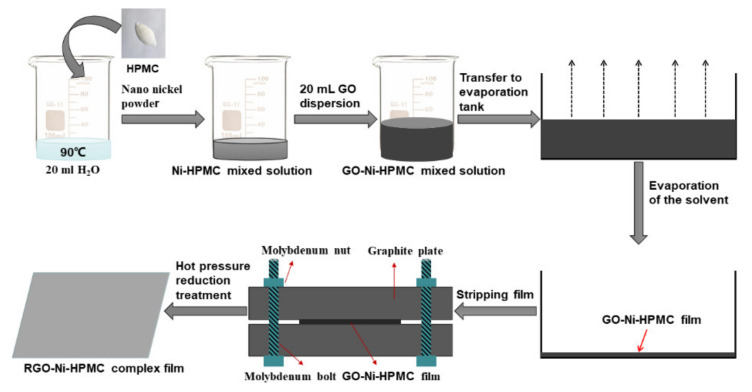
Flowchart of preparation of RGO-Ni-HPMC composite membrane.

The process chart for preparation of RGO-Ni-HPMC composite membrane is shown as following (Figure 1): (1) Add 20 mL of deionized water into a 50 mL beaker and heat it to 90 °C in a circulating water bath, then add 10 mg of HPMC and stir it until fully dissolving to prepare HPMC solution. (2) After the solution of HPMC was cooled down to room temperature, 15 mg nano Ni powder was added into it and subjected to ultrasonic treatment for 2 min, and then nano Ni powder was surrounded by HPMC molecules and uniformly dispersed in the solution (Figure S1, Supplementary Materials). (3) First, 20 mL GO dispersion (5 mg/mL) was ultrasonically treated for 10 min, and then was added into the mixture of hydroxypropyl methyl cellulose-Ni powder and stirred evenly with glass rod to obtain the GO-Ni-HPMC mixture. (4) The mixture of GO-Ni-HPMC was transferred to a 5 cm × 10 cm evaporation tank with organic glass plate as the substrate, and dried at 60 °C for 8 h to obtain GO-Ni-HPMC film, which was denoted as GO-Ni-HPMC-15, with a thickness of 34 μm; (5) remove GO-Ni-HPMC membrane from the substrate and of rolling press it with a roller, and then load it in the hot-pressing mold, after that thermally reduce it in the high temperature tube furnace equipment (Shanghai Victory Instrument Co., Ltd. Shanghai, China. SLG1200-100) with the sample of hot-pressing mold under pressure around 8 mPa, during which the temperature is raised from room temperature to 300 °C under the protection of argon at a 10 °C/min heating rate, then to 800 °C at a 3 °C/min heating rate and maintain for 2 h. Finally, RGO-Ni-HPMC composite film was obtained after natural cooling to room temperature (cooling time is about 3 h) under the protection in argon atmosphere, which is denoted as RGO-Ni-HPMC-15 composite film with a thickness of 26 μm. Similarly, keeping other conditions unchanged, change the amount of nano Ni powder added as 0 mg, 5 mg, 10 mg and 20 mg, and denoted as RGO-Ni-HPMC-0, RGO-Ni-HPMC-5, GO-Ni-HPMC-10 and RGO-Ni-HPMC-20 composite membrane. In particular, when the amount of added nano Ni powder and HPMC is 0 mg, the prepared RGO film is denoted as RGO film. When the addition amount of nano Ni powder is 15 mg and HPMC is 0 mg, the prepared sample is denoted as RGO-Ni-15 composite film.

### 2.3. Representation

In this study, the following characterization analysis and tests were carried out:

The surface morphology and cross section of the thin film samples were observed with a super high resolution field emission electron microscope (Hitachi, Tokyo, Japan, equipment: Hitachi Uhr FE-SEM SU8220) at an accelerating voltage of 10.0 kV. The chemical bonds and element contents of the samples were analyzed with photoelectron spectroscopy (XPS) (Kratos, Axis Ultra DLD, Manchester, England). The sample and KBr powder were mixed and pressed into transparent film, and the functional group changes of the sample before and after thermal reduction were analyzed with Fourier transform infrared spectrometer (FTIR, Tensor 27, Bruker, Bremen, Germany).

With Alpha ray as a radiation source, The test is conducted using Cu target K in wavelength of 0.15406 nm, test voltage for 40 kV, and tube current of 40 ma of samples under the condition of X ray diffraction (XRD, D8 Advance, AXS, Karlsruhe, Germany), and according to the Bragg equation the experiment sample material interplanar spacing is calculated, the Bragg equation: 2dsinθ = nλ, among which, the d as spacing, θ as the angle among the incident ray, the reflected ray and the reflective crystal, and λ as wavelength (λ = 0.15406 nm), n as reflection series.

### 2.4. Thermal Conductivity Test

The planar thermal diffusivity of all samples was measured at room temperature (25 °C) using a laser flash (Netzsch LFA 447, Selb, Germany), a method reported in our previous work [13]. The thermal conductivity (*λ*) can be calculated by Equation (1):

(1) $$\lambda =\alpha \cdot \rho \cdot C_{p}$$

*λ* is the thermal conductivity of the sample (W/(m·K)); *α* represents the thermal diffusivity (m^2^/s) of the sample; ρ is the density of the sample (g/cm^3^), ρ=m/V formula calculated, where m is the mass of the film sample, the use of 1/10,000 electronic balance weighing obtained, V is the volume of the film sample, the volume of the film sample V = πR^2^d/4, R is the sample diameter, d is the sample thickness. $C_{p}$ is the constant pressure specific heat capacity of the sample (J/(g·K)). Differential scanning calorimetry (DSC; Netzsch DSC 204 F1, Netzsch, Selb, Germany) was tested under the scanning temperature condition of −10–50 °C.

A thermal test platform was established. The center of the sample was radiated by ceramic rod heating probe and the surface temperature distribution of the sample was recorded by an infrared camera. A test platform was established to simulate the heat dissipation of electronic equipment. The samples were heated at a constant temperature with a constant temperature heating pad, and an infrared camera (FLIR MSX Technology, FLIR, Boston, MA, USA) was used to characterize the heat dissipation efficiency of the samples against high-temperature components and record the temperature distribution, so as to verify the heat conduction effect of the samples in the plane.

## 3. Results

The heat conduction of graphene-based carbon materials is mainly dependent on phonon transport (i.e., lattice thermal vibration). Compared with small-sized graphene, large-sized graphene has larger phonon transport free path and lower interfacial phonon scattering so that it is considered more suitable for assembling graphene-based film with high thermal conductivity [14,15]. In the process of preparing GO with Hummers’ method, the size of GO sheet and oxidant have a direct influence on the degree of natural graphite oxidation. The lattice structure of GO obtained with moderate graphite oxidation degree is relatively complete, and it is easy to obtain large size GO sheet after ultrasonic stripping [16,17]. Using potassium permanganate as the oxidants, this experiment adopts low oxidant/graphite mass ratio (graphite: k = 1:3), and by controlling the degree and depth of graphite oxidation large-sized. GO sheet is obtained with transverse size as large as in the range of 5.0–20 μm (Figure S2, Supplementary Materials). Besides its lattice structure remains relatively complete with good dispersion capability in aqueous solution (Figure S3, Supplementary Materials), and therefore it is suitable for the assembly of graphite heat conduction membrane materials.

**Figure 2:**
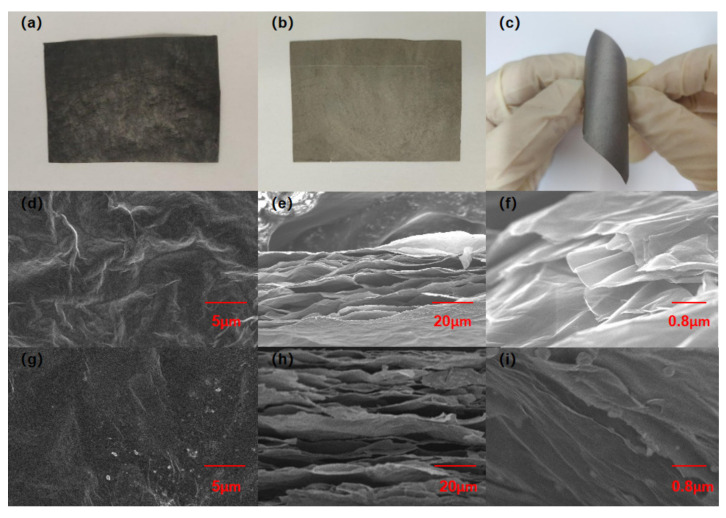
(**a**) Optical diagram of GO-Ni-HPMC-15 film; (**b**) Optical diagram of RGO-Ni-HPMC-15 composite film; (**c**) RGO-Ni-HPMC-15 composite film bending folding optical diagram; (**d**) Surface microstructure of RGO film; (**e**,**f**) RGO film cross section microstructure diagram; (**g**) Surface microstructure of RGO-Ni-HPMC-15 composite membrane; (**h**,**i**) RGO-Ni-HPMC-15 composite membrane cross section microstructure diagram.

The RGO film and RGO-Ni-HPMC-15 carbon based composite film were prepared by solvent evaporation and hot pressing reduction processes using the prepared large-size GO as the main raw material, HPMC as the external solid carbon source, and Ni nanoparticles as the catalyst. The macroscopic performance and microstructure of the films are shown in Figure 2. Figure 2a is the optical diagram of GO-Ni-HPMC-15 film. It is black in color with a slightly coarse surface, and can be folded for many times without cracks. As shown in Figure 2b,c, the RGO-Ni-HPMC-15 composite film treated with hot pressing reduction process is gray in color with a flat surface. The film has neither cracks when folded, and even no cracks appear after 1000 bending cycle tests (Figure S4, Supplementary Materials), which demonstrates good flexibility of RGO-Ni-HPMC-15 composite film. The reason why the RGO-Ni-HPMC-15 composite film still has good flexibility after the hot compression reduction process is that there is a strong van der Waals force between graphene sheets [18], which maintains the overall mechanical properties of RGO-Ni-HPMC-15 composite film. In terms of microstructure, SEM characterization is demonstrated in Figure 2d,e that the surface of RGO film is wrinkled, with obvious and deep gullies, good order between lamellae, general compaction and more obvious air holes. Furthermore, there is no granular object on the surface of the lamellae observed by SEM characterization in Figure 2f with magnification. Figure 2g,h shows the micro structure for RGO-Ni-HPMC-15 composite membrane, compared with RGO membrane, its surface is flat and shrivel and presents complete surface with some ripples and dense-packed layers. The reason for this is that Ni nanoparticles wrapped in HPMC scatter in graphene oxide layers and construct the GO-Ni-HPMC-15 gas discharge channels between membrane layers so that it is helpful for the discharge of CO_2_, H_2_O produced in thermal reduction process and the formation of smooth layers. In addition, from the Figure 2i magnification observation, spherical particles of various sizes can be seen on the surface of the layers. It is conjectured that they are Ni nanoparticles wrapped in multiple layers of graphene from the thermal reduction reaction. This is because Ni nanoparticles have small size, large specific surface area and large amount of surface defects so that they have strong adhesion and chemical reactivity. When HPMC forms O–Ni–O coordination covalent bond with Ni nanoparticles in the interaction, HPMC is adhered on the surface of Ni nanoparticles and wrapped them [19]. In the thermal reduction process of GO-Ni-HPMC-15 to form RGO-Ni-HPMC-15, methane and amorphous carbon generated by thermal decomposition of HPMC produce carbon atom through the catalysis of Ni nanoparticles which dissolve and diffusive into the Ni particles [20], and further precipitate multiple layers of graphene on the surfaces of Ni nanoparticles in the process of cooling due to the supersaturation, namely the spherical particles observed in the SEM Figure 2i. Since the heat transfer of graphene is achieved by phonon transport, the phonons diffuse from the high concentration region to the low concentration region and transfer the heat in a diffusion manner [21]. Therefore, the graphene-based membrane structure with smooth surface, orderly lamellar arrangement and densely packing is more conducive to reducing the collision probability between phonons and thus improving the overall heat conduction efficiency of the graphene-based membrane.

**Figure 3:**
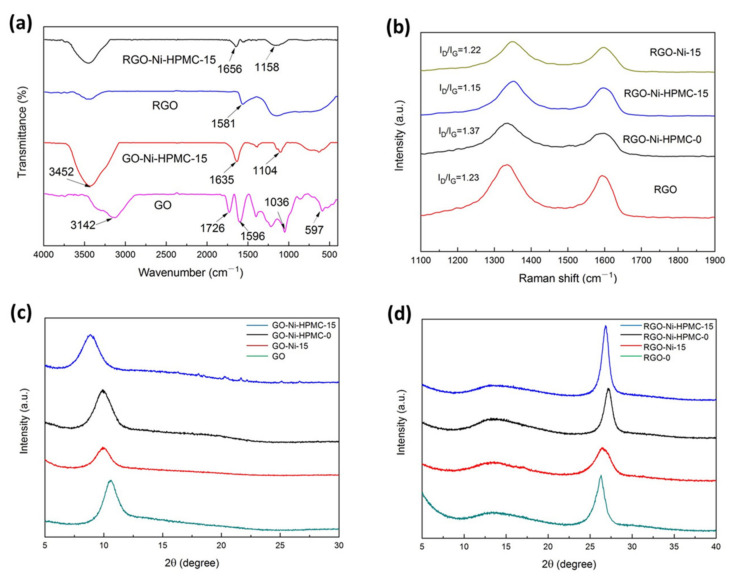
(**a**) Shows the infrared spectra of GO and GO-Ni-HPMC-15 films before and after thermal reduction treatment; (**b**) Raman spectra of RGO-Ni-HPMC-15, RGO-Ni-HPMC-0, RGO and RGO-Ni-15 films; (**c**) XRD images of GO-Ni-HPMC-15, GO-Ni-HPMC-0, GO and GO-Ni-15 film; (**d**) XRD analysis of RGO-Ni-HPMC-15, RGO-Ni-HPMC-0, RGO and RGO-Ni-15 film.

As shown in Figure 3a, infrared spectra demonstrates the composition and structure changes of GO and GO-Ni-HPMC-15 films before and after thermal reduction. Before carbon thermal reduction process, carboxyl on GO, hydroxyl and combined water between the layers exists in associative state. On the spectrum figure, a wide –OH stretching vibration mode is present at 3142 cm^−1^, at 1382 cm^−1^ a weaker bending vibration peak is shown [22], and relatively sharp stretching vibration absorption peaks are shown at 1726 cm^−1^, 1596 cm^−1^, 1036 cm^−1^ for C=O, C=C-, C–O–C group, respectively [23]. Since HPMC and bound water both contain abundant oxygen-containing groups such as –OCH_2_CHOHCH_3_ and hydroxyl, the oxygen-containing groups form stable O–Ni–O coordination covalent bond with Ni nanoparticles through mutual covalent interaction, which is strongly affected by the electron-absorbing action of metal Ni atoms. Therefore, the hydroxyl characteristic peak of GO-Ni-HPMC-15 composite film showed obvious blue shift, and showed a wide stretching vibration mode at 3452 cm^−1^ in spectrum figure. In addition, the HPMC hydroxypropyl oxygen radicals (–OCH_2_CHOHCH_3_) and methoxy (–OCH_3_) have strong infrared absorption. Therefore the absorption peaks of other oxygen-containing groups on the graphene oxide are covered so that the GO-Ni-HPMC-15 composite membrane only shows more sharp stretching vibration absorption peaks at 1635 cm^−1^ and 1104 cm^−1^, respectively for the C=C and C–O–C group [23], and methyl on HPMC only shows a weaker stretching vibration peak at 1388 cm^−1^. After thermal reduction treatment at 800 °C, the oxygen-containing functional groups of the GO-Ni-HPMC-15 membrane were basically removed, leaving an obvious C=C stretching vibration characteristic peak at 1656 cm^−1^, indicating that the RGO-Ni-HPMC-15 composite membrane is mainly composed of carbon-carbon double bond structure. In addition, the RGO-Ni-HPMC-15 composite membrane also shows a hydroxyl stretching vibration peak near 3434 cm^−1^ in the spectrum figure, which is caused by the absorption of a small amount of water in the air during the sample preparation process.

Raman spectroscopy is an important means to analyze the structural defects of carbon materials. The number of layers, defects and crystal structure of graphene materials are characterized according to the intensity, peak position and half-peak width of Raman spectroscopy [24]. The Raman spectra of graphene are mainly composed of two high intensity D peak and G peak, of which D peak is considered as the disorder oscillation peak of graphene and used to characterize the structural defects and edges of graphene materials, while G peak is considered as the main characteristic peak of graphene, caused by the planar vibration of sp^2^ hybrid carbon atoms [25]. The defect density of graphene can be characterized according to the intensity ratio of peak D and peak G. The smaller the intensity ratio of peak D and peak G is, the smaller the defect density of graphene is [26]. According to Figure 3b, two characteristic peaks of RGO film can be observed, which are D peak near 1350 cm^−1^ and G peak near 1580 cm^−1^, respectively, and their I_D_/I_G_ intensity ratio is 1.23. When HPMC modification reagents were added, the I_D_/I_G_ of RGO-Ni-HPMC-0 composite membrane was 1.37, and the defect density increased compared with RGO membrane, which was caused by the thermal decomposition of HPMC to produce amorphous carbon substances. The I_D_/I_G_ strength of the RGO-Ni-15 composite membrane with only Ni nanoparticles added is 1.22, which is similar to the I_D_/I_G_ strength of the RGO membrane. This is because the Ni nanoparticles have no repair effect on the structural defects of graphene, so the defect density of graphene does not change much. When the mass ratio of GO, Ni and HPMC is 100:15:10, the I_D_/I_G_ of the RGO-Ni-HPMC-15 composite membrane is 1.15, which significantly reduces the I_D_/I_G_ of the RGO-Ni-HPMC-0 composite membrane, that is, the defect density is greatly reduced. This is because the Ni nanoparticles catalyze the pyrolysis of methane and amorphous carbon generated by the thermal decomposition of HPMC, resulting in the dissolution of carbon atoms into the interior of the Ni particles. Later, due to supersaturation, carbon atoms dissolved in the cooling process precipitate carbide with multilayer graphene structure on the surface of Ni particles. The defect density of carbide with multilayer graphene structure is smaller than that of RGO film, so the overall defect density of RGO-Ni-HPMC-15 composite film is smaller. Since the heat transfer of nonmetallic materials is realized through phonon transmission, the existence of defects will become the phonon scattering center and decrease the thermal conductivity of graphene-based film. When the defect density is reduced, the phonons are less affected by the scattering, and the heat transfer efficiency of graphene is higher. Therefore, the RGO-Ni-HPMC-15 composite membrane structure is more conducive to heat conduction in the plane direction.

XRD can be used for qualitative analysis of a substance according to the position of diffraction characteristic peak, and it is also an effective means to analyze the crystallinity and grain size of a substance. The diffraction peak of the original graphite appears at 26.45°, and the interplanar spacing is 0.337 nm according to the Bragg lattice equation (2dsinθ = nλ) (Figure S5, Supplementary Materials). The XRD spectrum of GO film is shown in Figure 3c. Its diffraction peak appears at the position of 10.6°, and its crystal plane spacing is calculated to be 0.834 nm. Compared with the original graphite, GO film has larger crystal plane spacing, which is due to the presence of bonded water and more oxygen-containing groups between GO sheets [27], which increases the crystal plane spacing of GO film. In addition, as shown in Figure 3c, the diffraction peaks of GO-Ni-15, GO-Ni-HPMC-0 and GO-Ni-HPMC-15 films appear at 10.10°, 9.92° and 8.81°, respectively, and the crystal plane spacing is 0.875 nm, 0.891 nm and 1.003 nm, respectively, indicating that the dispersion of Ni nanoparticles and HPMC between GO sheets increases the crystal plane spacing of GO films, and with the increase of doping amount, the crystal plane spacing of GO films increases. As shown in Figure 3d, after the thermal pressing reduction process, the diffraction peaks of RGO, RGO-Ni-15, RGO-Ni-HPMC-0 and RGO-Ni-HPMC-15 films appear at 26.16°, 26.45°, 27.2°and 26.82°, respectively. The positions of the diffraction peaks are all close to the characteristic peaks of the original graphite, indicating that the above samples all have the same graphene structure as the original graphite. Secondly, from the perspective of peak shape, the diffraction peak of RGO-Ni-HPMC-15 composite film is relatively narrow and high, which is speculated to be due to the catalytic cracking of Ni nanoparticles to generate carbides with multilayer graphene structure, thus increasing the grain size and crystal phase content of RGO-Ni-HPMC-15 composite film. In addition, the diffraction peaks of GO-Ni-15 and GO-Ni-HPMC-0 films appear at 26.45° and 27.2°, respectively, and the peaks are relatively wide and low, indicating that only doped Ni nanoparticles or HPMC have no significant effect on the crystal phase content and grain size of graphene. The structure of the RGO-Ni-HPMC-15 composite film is more conducive to heat conduction because the large size of grains is conducive to the phonon transmission within the graphene grain and the reduction of phonon scattering at the grain boundary [28].

**Figure 4:**
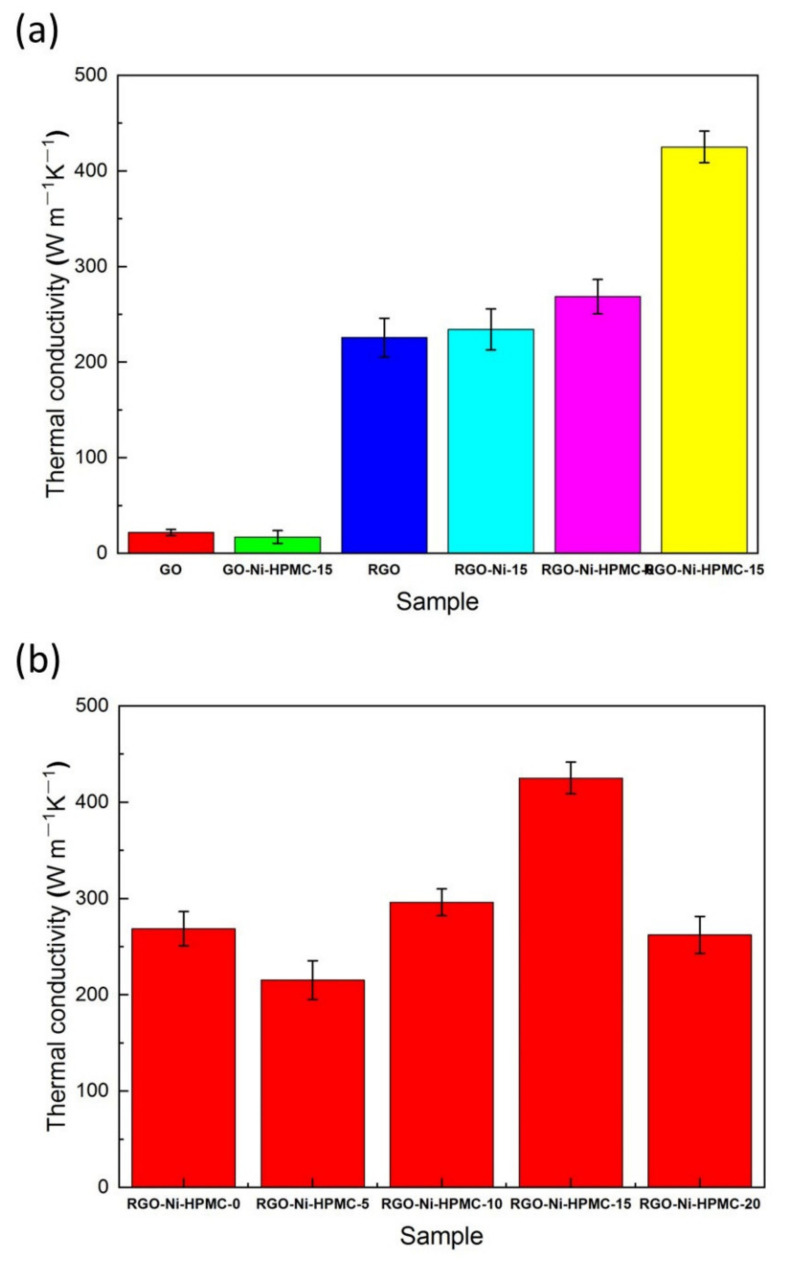
(**a**) Plane thermal conductivity of different types of carbon-based films; (**b**) Plane thermal conductivity of RGO-Ni-HPMC composite film.

The density and thickness of each sample for the laser flash method are listed in Tables S1 and S2 (Supplementary Materials). As shown in Figure 4a, the plane thermal conductivity of different types of carbon-based films was calculated by the thermal conductivity calculation Equation (1). At room temperature, the planar thermal conductivity of GO films without thermal reduction is 20.91 W/(m·K), and that of GO-Ni-HPMC-15 is 17.40 W/(m·K). This is because the oxygen-containing groups in the graphene oxide destroy the conjugated structure of graphene, making its thermal conductivity greatly reduced. The thermal conductivity of the RGO film obtained by the hot pressure reduction process is 225.7 W/(m·K). Due to the removal of the bound water and most oxygen-containing groups (such as epoxy group, hydroxyl group and carboxyl group) in the GO film, its conjugated structure is recovered to a large extent, thus the thermal conductivity of the RGO film is greatly improved. The thermal conductivity of RGO-Ni-15 and RGO-Ni-HPMC-0 composite films supplemented only with Ni particles or HPMC is similar to 234.2 W/(m·K) and 268.5 W/(m·K), respectively, indicating that the thermal conductivity of graphene based films doped only with Ni particles or HPMC has no significant change. Different from the RGO-Ni-15 composite film and the RGO-Ni-HPMC-0 composite film, the thermal conductivity of the modified RGO-Ni-HPMC-15 composite film is greatly improved. The plane thermal conductivity of the modified RGO-Ni-HPMC-15 composite film is 425.1 W/(m·K), which is 88.3% higher than that of the RGO film. This indicates that the combined application of Ni particles and HPMC can greatly improve the thermal conductivity of the graphene based film. This is because nano Ni particles adhered on the surface of the catalytic cracking the HPMC was generated with multilayer graphene structure of carbides, wrapped in layers of graphene Ni particles bridge in graphene layer between space and build the phonon transport channels so as to improve the overall heat transfer rate of the composite membrane. In addition, as shown in the Figure 4b, the thermal conductivity of the RGO-Ni-HPMC composite membrane is affected by the mass ratio of GO, Ni and HPMC. When the amount of GO and HPMC is constant, the thermal conductivity of the RGO-Ni-HPMC composite membrane increases first and then decreases with the increase of the addition amount of nano Ni particles. When the mass ratio of GO, Ni and HPMC is 100:15:10, the thermal conductivity of RGO-Ni-HPMC-15 composite film reached the maximum value. This is because the nano Ni particles larger than the surface area, surface atomic number, there are a large number of defects and suspension key, has a strong adsorption and chemical reactivity. Therefore, a moderate amount of doping nano Ni powder is helpful to realize the HPMC to Ni particles completely packages, and excess doped Ni nanoparticles cause HPMC particle to Ni package is insufficient. The surface defects of the Ni particles bare outside become phonon scattering center, which reduced the thermal conductivity of RGO-Ni-HPMC composite film.

**Figure 5:**
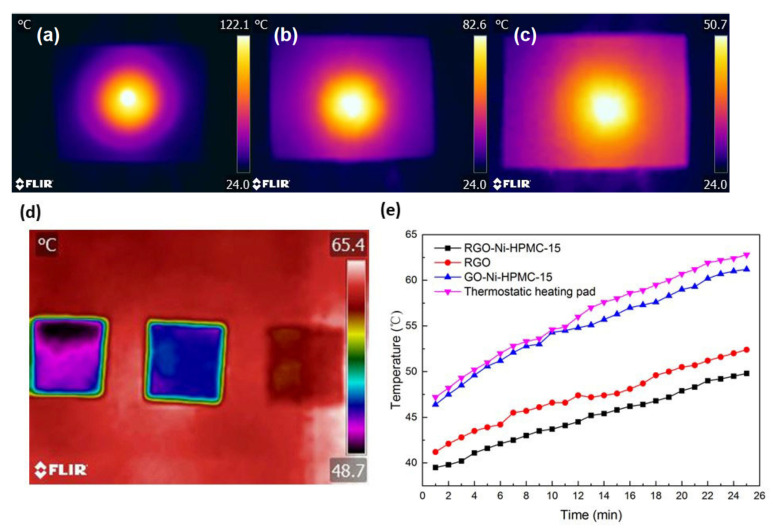
(**a**) Schematic diagram of thermal conductivity of GO-Ni-HPMC-15 composite film; (**b**) Schematic diagram of heat conduction performance of RGO film; (**c**) Schematic diagram of thermal conductivity of RGO-Ni-HPMC-15 composite film; (**d**) Heat dissipation diagram of analog electronic equipment; (**e**) Surface temperature and time variation of RGO-Ni-HPMC-15 composite film.

The heat dissipation principle of graphene-based membrane evenly distributes the heat at the hot spot on a two-dimensional plane and takes the heat away through the external cooling medium to ensure that the heating module works at the temperature it is subjected to. The thermal conductivity of GO-Ni-HPMC-15, RGO-Ni-HPMC-15 and RGO film was tested according to the test method of thermal conductivity and the test results are shown in the Figure 5. As shown in the Figure 5a, the GO-Ni-HPMC-15 composite film without thermal reduction process has poor heat conduction performance in the plane direction. The heat at the central heating position cannot be rapidly transferred to the edge of the film. The hot spot temperature is as high as 122.0 °C, while the temperature at the edge of the film is only 26.2 °C close to the ambient temperature. As shown in the Figure 5b, the hot spot temperature at the central heating position of the RGO film treated by the hot compression reduction process is 82.1 °C, while the temperature at the edge of the film is 33.8 °C, and its heat conduction performance in the plane direction is greatly improved. As shown in the Figure 5c, after Ni nanoparticles and HPMC modification of RGO-Ni-HPMC-15 composite membrane, highlights in the center of the heating temperature and edge temperature are 50.4 °C and 35.6 °C, respectively, direction of flat heat conduction performance is best, which can effectively avoid the phenomenon of local temperature is too high, the result and testing the GO-Ni-HPMC-15, RGO and RGO-Ni-HPMC-15 the plane thermal conductivity of membrane. As shown in the Figure 5d, the good heat dissipation and temperature control effect of RGO-Ni-HPMC-15 composite membrane was further verified by simulating the heat generating scene of electronic equipment through constant temperature heating pad. Figure 5e shows the surface temperature and time changes of the RGO-Ni-HPMC-15 composite membrane and the contrast material. The heating time of the thermostatic heating pad is from 0–25 min, and its surface temperature rises from 47.2 °C to 62.8 °C. The hot spot temperature of the corresponding RGO-Ni-HPMC-15 composite membrane temperature control part rises from 39.5 °C to 49.8 °C, which decreases by 11.5 °C on average, greatly reducing the working temperature of the heating module. In contrast, the surface temperature of the GO-Ni-HPMC-15 film without the hot-pressing reduction process is close to that of the thermostatic heating pad. However, the hot spot temperature of the temperature control part of the RGO film rises from 41.2 °C to 52.4 °C, which reduces the temperature of the thermostatic heating pad by 9.0 °C on average, and its heat dissipation effect is worse than that of the RGO-Ni-HPMC-15 composite film. Therefore, the RGO-Ni-HPMC-15 composite membrane prepared in this experiment can meet the basic requirements of high heat flux heat dissipation, and is expected to be applied in the field of thermal management on a large scale.

Graphene was prepared by chemical vapor deposition with Ni film as substrate. At high temperature (400–1000 °C), the gas carbon source precursor catalyzed cracking on the surface of Ni film to produce carbon atoms dissolved into Ni metal and diffused. When the temperature decreased, the dissolved carbon atoms precipitated on the metal surface due to supersaturation to form multilayer graphene [29,30]. Therefore, the growth of graphene can be realized by carburizing Ni particles to separate carbon. In this study, the GO-Ni-HPMC-15 membrane was prepared by solvent evaporation using GO as the main raw material, HPMC as the external carbon source and nano Ni particles as the catalyst. The GO-Ni-HPMC-15 membrane was annealed at 800 °C, and the methane and amorphous carbon generated by the thermal decomposition of HPMC were catalyzed by the nano-Ni particles to generate carbon atoms, which were dissolved into the Ni particles [20]. After that, the dissolved carbon atoms precipitated on the surface of the Ni particles due to supersaturation to generate carbides with multilayer graphene structure. Finally, Ni particles coated with multiple layers of graphene are bridged between graphene sheets and phonon transport channels are constructed through multiple layers of graphene to improve the overall heat transfer rate of graphene-based composite film.

## 4. Conclusions

In this study, a modest thermal reduction process was used to prepare graphene-based composite membrane. The prepared RGO-Ni-HPMC composite membrane maintained a high thermal conductivity and also had obvious low-cost advantages. Combined with the analysis of the test results, after modest hot-pressing reduction process, nano-Ni particles catalyzed cracking of the surface attached HPMC to generate multilayer graphene to construct phonon transport channels, thus improving the overall heat transfer rate of RGO-Ni-HPMC carbon-based composite film. When the mass ratio of GO, nano-Ni particles and HPMC is 100:15:10, the thermal conductivity of RGO-Ni-HPMC-15 composite film reaches the maximum, showing excellent plane thermal conductivity. Further simulation experiments of electronic equipment heat dissipation show that RGO-Ni-HPMC-15 composite film has a good heat dissipation effect on the hot spot at high temperature, and reduces the hot spot temperature by 11.5 °C on average. It is expected to solve the heat dissipation problem of high-power electronic equipment and be applied on a large scale in related thermal management fields.

## Data Availability

Please refer to Supplementary Materials for details of the manuscript.

## Supplementary Materials

The following are available online at https://www.mdpi.com/article/10.3390/nano11123392/s1, Figure S1: Ni-HPMC mixed solution, Figure S2: GO sheet transverse size, Figure S3: GO dispersions of different concentrations, Figure S4: Bending cycle test device, Figure S5: The XRD of original graphite, Figure S6: Heat generating scene of electronic equipment, Table S1: The thickness and the bulk density of samples in the LFA measurement, Table S2: The thickness and the bulk density of samples in the LFA measurement.
