# Supplementary material for: Graphene-Based Composite Membrane Prepared from Solid Carbon Source Catalyzed by Ni Nanoparticles
Source: Nanomaterials (Basel). 2021 Dec 14;11(12):3392. doi: 10.3390/nano11123392 (PMC8707696; doi:10.3390/nano11123392)
Supplement: Supplementary file 1 [file nanomaterials-11-03392-s001.zip › nanomaterials-1472752-supplementary.pdf]

nanomaterials

Supporting Information

Graphene-Based Composite Membrane Prepared from Solid Carbon Source Catalyzed by Ni Nanoparticles

Jing Li [1,2], Jialiang Liu [1], Jinshui Liu [1], Jinfeng Lai [1], Yuxun Chen [3,*] and Wenjun Li [4,*]

[1] School of Chemistry and Chemical Engineering, South China University of Technology, Guangzhou 510641, China; ljing@scut.edu.cn (J.L.); 201920122396@mail.scut.edu.cn (J.L.); 202020124091@mail.scut.edu.cn (J.L.); laijinfeng666@126.com (J.L.)
[2] SCUT-Zhuhai Institute of Modern Industrial Innovation, Zhuhai 519175, China
[3] School of Mechanical and Automotive Engineering, South China University of Technology, Guangzhou 510641, China
[4] Beijing headquarters of space vehicle, Beijing 100086, China
* Correspondence: yuxunch@scut.edu.cn (Y.C.); hitmmc@hotmail.com (W.L.)

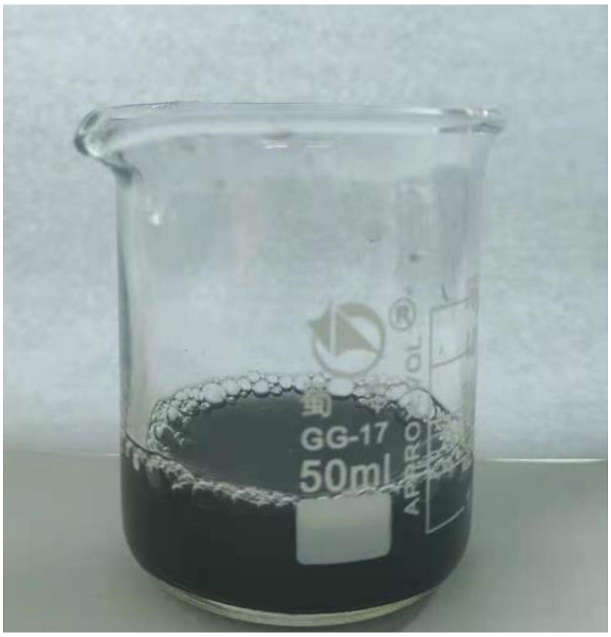

Figure S1. Ni-HPMC mixed solution.

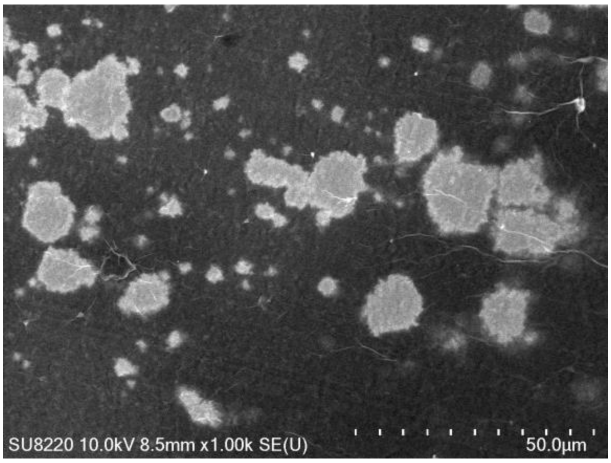

Figure S2. GO sheet transverse size.

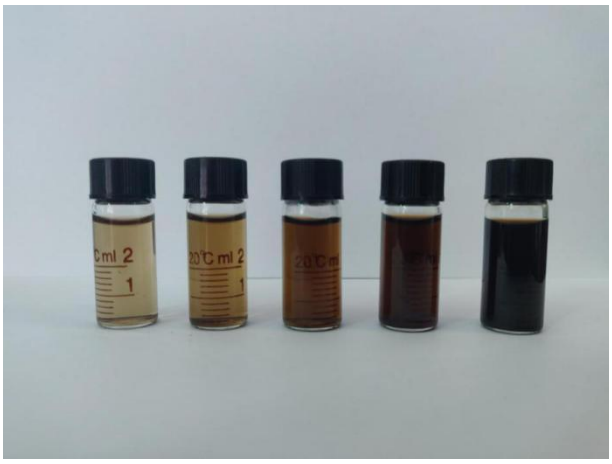

Figure S3. GO dispersions of different concentrations.

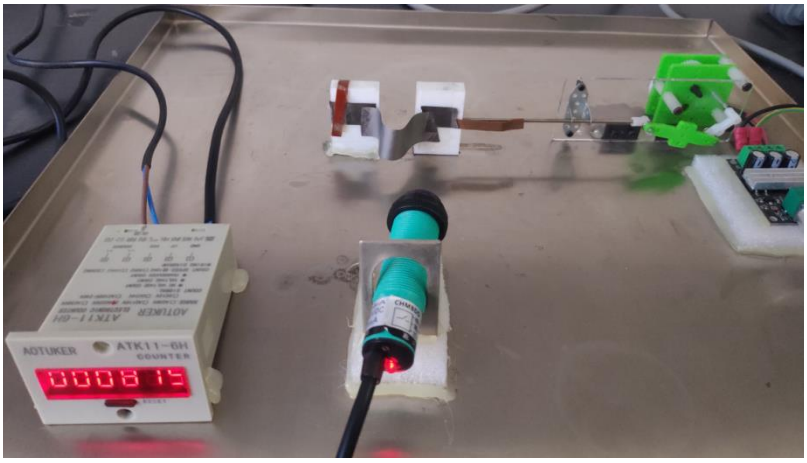

Figure S4. Bending cycle test device.

Figure S4, bending cycle test device consists of a reciprocating motion mechanism, a photoelectric counter and a sample table. The reciprocating motion mechanism consists of a small DC motor (rated voltage is 6 V) and a circuit protection device. Test samples in the process of the mechanical properties, shear into a specification for the sample of the first 3 cm × 5 cm of the strip shape with a transparent tape fixed between the fixed platform and slider, after starting power, reciprocating motion mechanism drawing slider do periodic reciprocating motion, every traction sliding block do reciprocating motion, a curved

circular slider drive samples, photoelectric counter samples according to the number of the slider block photoelectric sensor for bending cycle count. Finally, the number of bending cycles and the bending radius of the sample were recorded by camera video to comprehensively evaluate the mechanical properties of the sample.

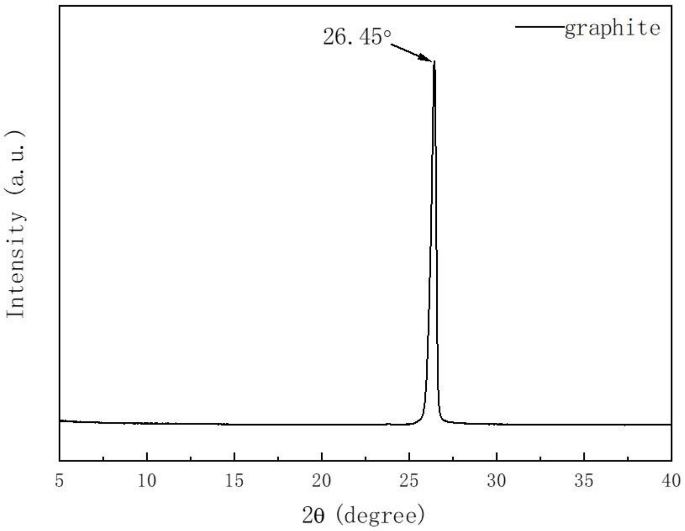

Figure S5. The XRD of original graphite.

Table S1. The thickness and the bulk density of samples in the LFA measurement.

Sample	Thickness (μm)	Bulk Density (g/cm^3)	Thermal Diffusivity (mm^2/s)	Specific Heat Capacity (W/(m·K))	Thermal Conductivity (W/(m·K))
GO	33	1.36	12.81	1.20	20.91
GO-Ni-HPMC-15	34	1.42	10.21	1.20	17.40
RGO	22	0.72	594.7	0.52	225.7
RGO-Ni-15	25	0.80	562.9	0.52	234.2
RGO-Ni-HPMC-0	25	0.76	679.4	0.52	268.5
RGO-Ni-HPMC-15	26	0.88	928.9	0.52	425.1

Table S2. The thickness and the bulk density of samples in the LFA measurement.

Sample	Thickness (μm)	Bulk Density (g/cm^3)	Thermal Diffusivity (mm^2/s)	Specific Heat Capacity (W/(m·K))	Thermal Conductivity (W/(m·K))
RGO-Ni-HPMC-0	25	0.76	679.4	0.52	268.5
RGO-Ni-HPMC-5	26	0.82	504.9	0.52	215.3
RGO-Ni-HPMC-10	28	0.86	662.2	0.52	296.1
RGO-Ni-HPMC-15	26	0.88	928.9	0.52	425.1
RGO-Ni-HPMC-20	25	0.90	928.9	0.52	262.2

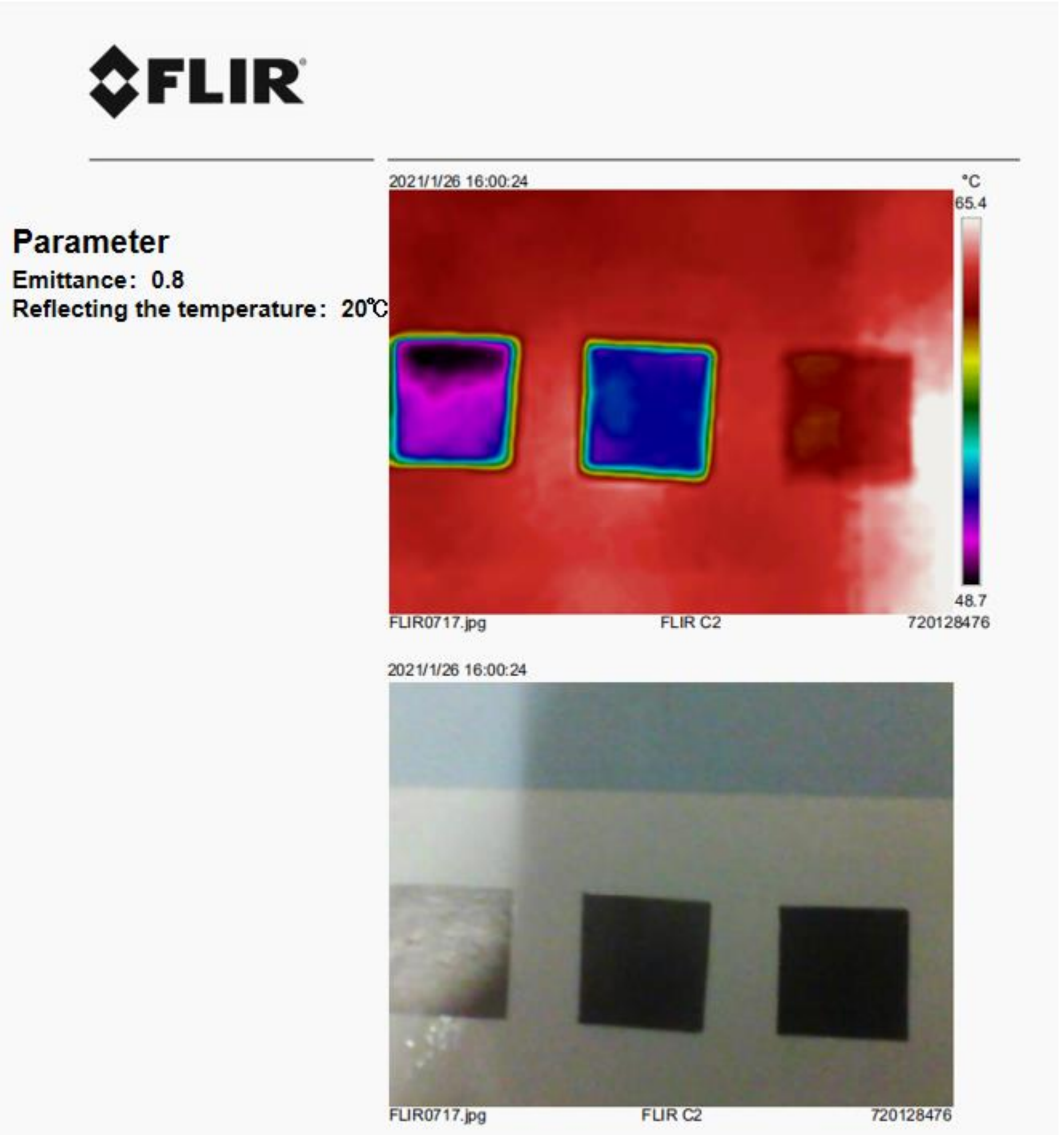

Figure S6. Heat generating scene of electronic equipment.
